# Relationship between hepatocellular carcinoma and depression via online database analysis

**DOI:** 10.1080/21655979.2021.1921552

**Published:** 2021-05-07

**Authors:** Tiantian Han, Yingchun Zhou, Danhua Li

**Affiliations:** a The First Clinical Medical College, Guangzhou University of Chinese Medicine, Guangzhou, China; b Clinical laboratory, The First Affiliated Hospital of Guangzhou University of Chinese Medicine, Guangzhou, China

**Keywords:** Hepatocellular carcinoma, depression, text mining, cell death or apoptosis

## Abstract

There may be a mutually reinforcing relationship between hepatocellular carcinoma (HCC) and depression, but the mechanism is unknown. This study used bioinformatics to evaluate the relationship between HCC and depression at the genetic level. Genes associated with HCC and depression were obtained from pubmed2ensemble. Overlapping genes were annotated by gene ontology (GO) function and enriched by Kyoto Encyclopedia of Genes and Genomes (KEGG) signal pathway. The cluster-1 genes obtained by Cytoscape were analyzed by GEPIA for expression and overall survival in HCC and, finally, introduced target genes to DGIdb to get associated drugs. A total of 199 genes were found to be in common between HCC and depression. GO term enrichment analysis on DAVID found the top-6 biological processes to be mainly associated with cell death and apoptosis. The top-6 cellular component terms are extracellular. The top-6 of molecular function terms are mainly associated with receptor binding. The top-6 pathways enriched by KEGG are mainly related to inflammatory response. IGF1, VEGFA, and SERPINE1 had statistical differences in expression and 10-year survival rate. There are total 45 drugs that act on VEGFA and SERPINE1. Based on our findings, we hypothesize that the mechanism of the interaction between HCC and depression may be related to cell death or apoptosis. Further studies are needed to verify this hypothesis.

## Graphical abstract

**Figure:**
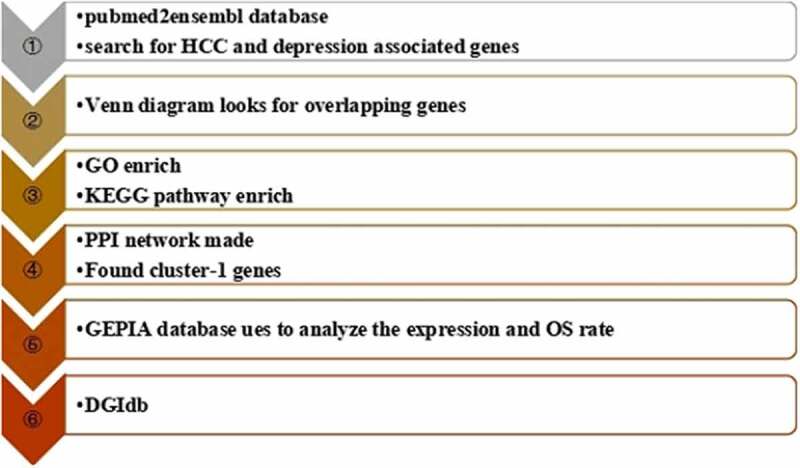

## Introduction

Hepatocellular carcinoma is the third most common cause of cancer deaths worldwide [1]. Due to various factors, including economic development and environmental factors, incidence is rapidly increasing across all age groups [2]. Hepatitis B, hepatitis C, aflatoxin exposure, alcohol consumption, and smoking are HCC risk factors [3]. Recent studies suggest that HCC is also associated with emotional and psychological factors. Numerous studies have reported high depression incidence in cancer patients. A Taiwanese prospective study involving 128 newly diagnosed HCC patients found that advanced Barcelona Clinic liver cancer stage, radiofrequency ablation therapy, or liver resection was most closely associated with worsening physical and psychological symptoms over time [4]. Anxiety disorders and depression have also been associated with a significantly increased overall survival rate in HCC patients in a retrospective study involving 7304 patients treated for HCC from 1996 to 2010 [5]. Thus, clinical professionals should pay attention to physical and psychological factors during treatment.

Depression is characterized by physical symptoms like fatigue, pain, or sleep disorder. Symptoms of depression may or may not occur [6]. Numerous studies have associated depression with a variety of chronic diseases, including cancer, coronary heart disease, and diabetes. The relationship between depression and cancer risk is characterized by two aspects: on one hand, depression may increase risk of some cancers [7,8] and, on the other, depression and other mental disorders may accelerate deterioration in cancer patients. It is reported that somatic depressive symptoms are similar in depression cancer patients and depression patients without chronic somatic symptoms and that depression cancer patients show greater physical depression symptoms than depression patients without chronic somatic symptoms [9]. The emergence of high levels of somatic symptoms should prompt clinicians to investigate potential complications in cancer patients. Due to the high prevalence of depressive symptoms and their impact on survival, psychological and drug intervention should be designed and implemented for patients diagnosed with liver cancer [10]. Thus, further study of relationship between HCC and depression will greatly improve prevention and treatment of the disease.

Advances in information technology and interdisciplinary research have led to increased use of bioinformatics in clinical research. For example, with the rapid development of sequencing technology, the field of gene detection has been redefined based on the next-generation sequencing (NGS) and due to the complexity of resulting datasets, bioinformatics has become a necessary tool in clinical NGS testing [11]. As for the treatment of cancer, more and more patients are being targeted by the detection of drug resistance genes. Thanks to the development of bioinformatics, researchers can design more small molecule targeted drugs to target gene therapy.

The potential mechanisms underlying HCC and depression are unknown. Here, we applied bioinformatics to identify common genes between HCC and depression. Genes associated with HCC and depression were identified on Pubmed2ensemble and those in common between the two subjected to GO and KEGG pathway analyses. PPI network analysis was used to identify core gene clusters, in order to provide more evidence for a relationship between HCC and depression at the genetic level.

## Materials and methods

### Acquisition of HCC and depression genes

Pubmed2ensemble data sources (http://pubmed2ensembl.ls.manchester.ac.uk/) are a customized and extended version of the Ensembl BioMart on genes. Input our target disease ‘hepatic carcinoma’ and ‘depression’ separately, choose ‘retrieve up to 100,000 document IDs, and select Homo sapiens genes (GRCh37) to get the gene set of related keywords.

### Acquisition of overlapping genes between HCC and depression

Through the online tool: Venny2.1 (https://bioinfogp.cnb.csic.es/tools/venny/), genes related to HCC were put into List1, while related genes of depression were filled in List2. Then, we acquired the overlapping genes between them.

### Functional and signal pathway enrichment analysis

Intersection gene obtained by Venny was introduced into DAVID 6.8 (https://david.ncifcrf.gov/), The Database for Annotation, Visualization and Integrated Discovery and analyzed using the parameters: OFFICIAL-GENE-SYMBOL, species: Homo sapiens. GOTERM_BP_FAT, GOTERM_CC_FAT, and GOTERM_MF_FAT were downloaded after GO and KEGG_PATHWAY analyses. BP, CC, and MF refer to biological process, cellular component, and molecular function, respectively.

### Protein–protein interaction (PPI) analysis and gene module analysis

Intersection genes were analyzed on STRING (https://string-db.org) to create an interaction network using the settings: multiple proteins, species: Homo sapiens, minimum required interaction score: 0.900, hide disconnected nodes in the network, and download PPI network from TSV file. The TSV file was imported into the Cytoscape, and the MCODE plugin was used to analyze core functional groups of the PPI network. MCODE can be used to construct functional modules in large gene (protein) networks. Cluster-1, obtained after clustering, had 26 core genes.

### Expression of cluster-1 gene in HCC and its effect on survival rate

The 26 genes were inputted into GEPIA (http://gepia.cancer-pku.cn/), which can analyze the expression of related genes in specific tumors and their effects on survival rate. First, we analyzed expression of the 26 genes in HCC and set the parameters: Log2FC cutoff: 1; p-value cutoff: 0.01. Then, we analyzed overall survival, group cutoff selected median, cutoff-high (%): 50; cutoff-low (%): 50. Expression of the genes was shown in boxplots and survival plots for HCC. Target genes for next analysis were brought together, which were both statistically significant between expression and survival rates.

### Drug screening

Genes with statistically significant expression differences and genes with statistically significant survival rates were introduced into DGIdb (https://www.dgidb.org/search_interactions), which was used to search for drug–gene interactions. Entered the target genes, IGF1, VEGFA, and SERPINE1, preset Filters selected “Approved”.

## Results

This study aimed to uncover the possible mechanism underlying the interaction between HCC and depression using bioinformatics. From our findings, we hypothesize that interaction between the two may exist in cell death/apoptosis. We also searched for drugs that can act on core genes and which may guide further research.

### Common gene in HCC and depression

Through the gene2ensemble database, 2586 HCC-associated genes and 382 depression-associated genes were identified, with 199 genes common between HCC and depression.

### Functional and signal pathway enrichment analysis

**Figure 1.**
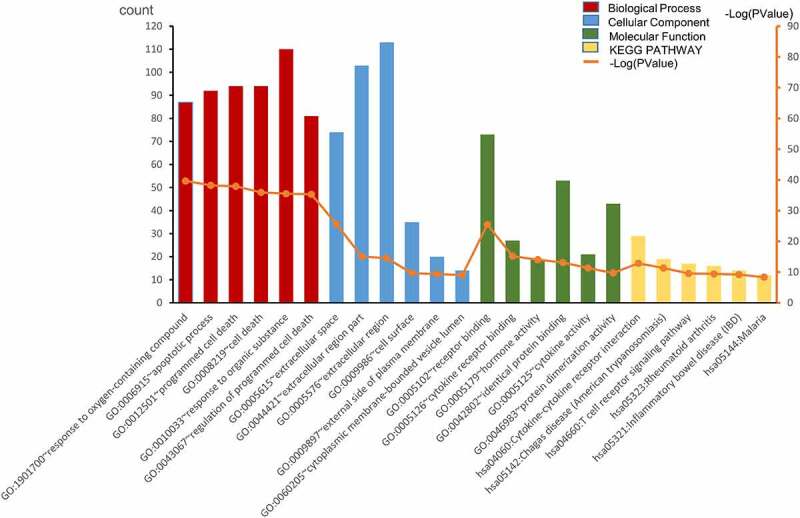
The top-6 of GO enrichment and KEGG pathway enrichment. Count means how many genes fall on the biological function/signal pathway. -Log(PValue): calculate the -Log10 value of *P* value for easy display in the figure

Based on GO enrichment analysis on DAVID, the top-6 biological processes were response to oxygen-containing compound, apoptotic process, programmed cell death, cell death, response to organic substance, and regulation of programmed cell death. The top-6 cellular component terms were extracellular space, extracellular region part, extracellular region, cell surface, external side of plasma membrane, and cytoplasmic membrane-bounded vesicle lumen. The top-6 molecular function terms were receptor binding, cytokine receptor binding, hormone activity, identical protein binding, cytokine activity, and protein dimerization activity. The top-6 KEGG pathways were cytokine–cytokine receptor interaction, Chagas disease (American trypanosomiasis), T cell receptor signaling pathway, rheumatoid arthritis, inflammatory bowel disease (IBD), and malaria (Figure 1).

### Protein–protein interaction analysis and gene module analysis

**Figure 2.**
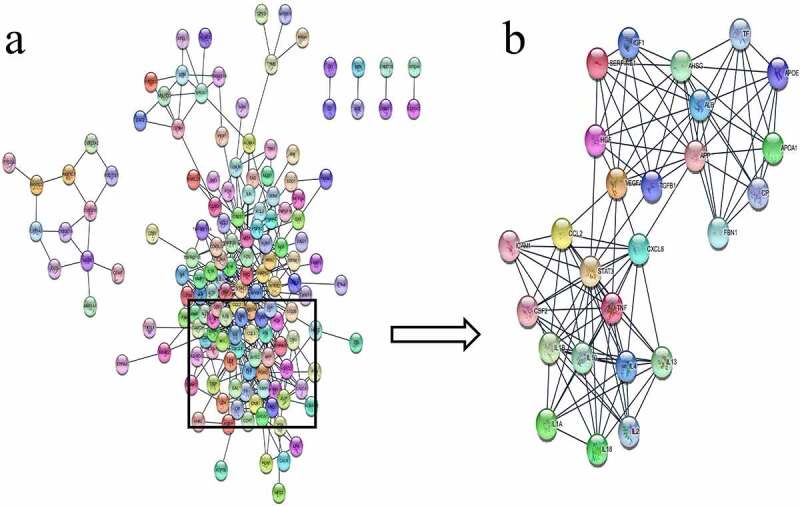
PPI network. (a): The 199 common genes. (b) Network of cluster-1 gene

STRING analysis of the 199 common genes identified a PPI network comprising 148 points and 473 edges (Figure 2(a)). In this network, the most prominent gene modules, cluster-1, was clustered using MCODE plugin on Cytoscape, with a total of 26 genes (Figure 2(b)).

### Expression of cluster-1 genes in HCC and its effect on survival rate

**Figure 3.**
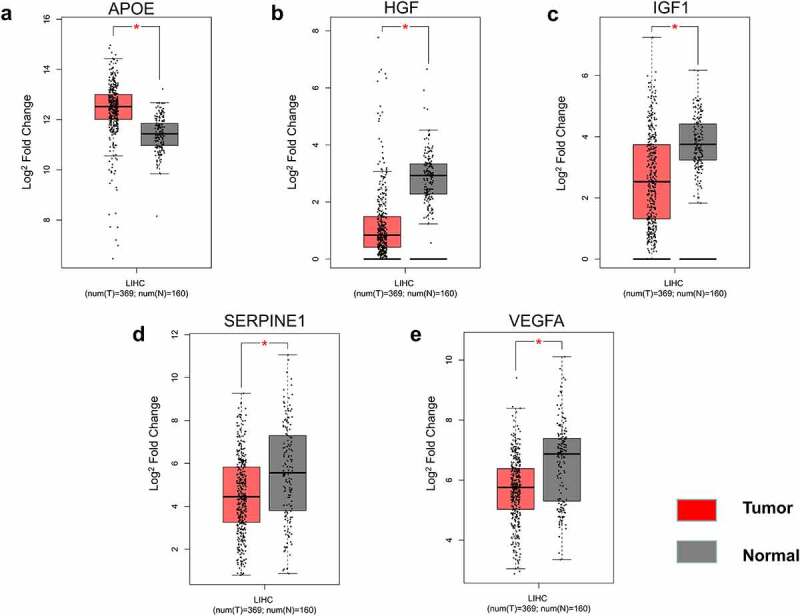
Gene expressed in hepatocellular carcinoma group and normal group. Validation of the gene expression of APOE (a), HGF (b), IGF1 (c), SERPINE1 (d), and VEGFA (e). The cutoff was set to |log2 fold change (FC)| ≥1, and *p* < 0.01. *p < 0.01

**Figure 4.**
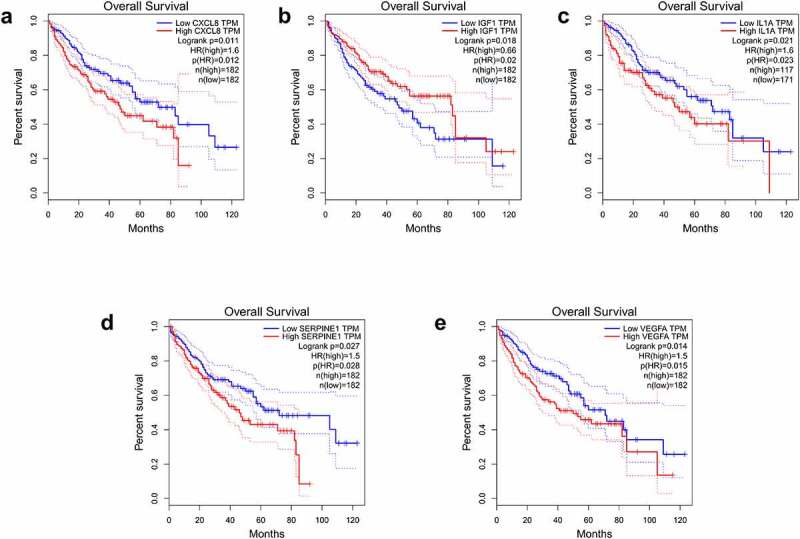
Overall survival analysis of the expression level of IGF1, IL1A, CXCL8, VEGFA, and SERPINE1 in hepatocellular carcinoma on GEPIA website. CXCL8 (a), IGF1 (b), IL1A (c), SERPINE1 (d), and VEGFA (e)

The 26 genes in cluster-1 were individually subjected to GEPIA analysis and disease was selected as liver hepatocellular carcinoma. This analysis identified IGF1, APOE, HGF, VEGFA, and SERPINE1 as differentially expressed in the HCC vs normal group (Figure 3), with IGF1, HGF, VEGFA, and SERPINE1 poorly expressed and APOE highly expressed. The differences in 10-year survival rates were IGF1, IL1A, CXCL8, VEGFA, and SERPINE1 (Figure 4). Among them, IGF1, VEGFA, and SERPINE1 had statistical differences in both expression and 10-year survival rate.

### Drug selection

Through the DGIdb database, we screened 45 drugs for interaction with IGF1, VEGFA, and SERPINE1. No drugs with interaction with IGF1 were identified, while 26 drugs interact with VEGFA and 19 with SERPINE1 (Table 1).

**Table 1.** Drugs acting on VEGFA and SERPINE1

Number	Drug	Gene	Score	Pubmed ID
1	Vandetanib	VEGFA	0.08	None found
2	Ranibizumab	VEGFA	23.96	18046235
3	Pegaptanib sodium	VEGFA	8.71	23953100
4	Carboplatin	VEGFA	0.11	26194361
5	Sildenafil	VEGFA	0.67	23007311
6	Lenalidomide	VEGFA	0.27	None found
7	Fenofibrate	VEGFA	0.28	11356390
8	Cisplatin	VEGFA	0.06	24090479
9	Phenytoin	VEGFA	0.21	15458527
10	Irinotecan	VEGFA	0.25	20125120
11	Minocycline	VEGFA	1.02	11875741
13	Bevacizumab	VEGFA	2.23	18182667
14	Oxaliplatin	VEGFA	0.47	24090479
15	Cilostazol	VEGFA	0.67	11524048
17	Fluorouracil	VEGFA	0.17	2400479
18	Enalapril	VEGFA	0.44	26002049
19	Gliclazide	VEGFA	3.11	17602961
20	Regorafenib	VEGFA	0.16	None found
21	Sunitinib	VEGFA	0.2	22948895
22	Sorafenib	VEGFA	0.2	22736425
23	Gentamicin	VEGFA	0.38	14986132
24	Capecitabine	VEGFA	0.36	20125120
25	Docetaxel	VEGFA	0.21	25111199
26	Leucovorin	VEGFA	0.47	20125120
27	Anistreplase	SERPINE1	1.4	17963464
28	Alteplase	SERPINE1	0.2	17963464
29	Orlistat	SERPINE1	0.42	11410822
30	Epirubicin	SERPINE1	0.14	16444429
31	Fibrinolysin, human	SERPINE1	0.35	None found
32	Citalopram	SERPINE1	0.12	18794724
33	Tenecteplase	SERPINE1	0.7	14564902
34	Reteplase	SERPINE1	1.4	17963464
35	Nimodipine	SERPINE1	0.2	11486117
36	Urokinase	SERPINE1	1.47	12709915
37	Copper	SERPINE1	0.03	21280128
38	Captopril	SERPINE1	0.25	9152782
39	Fluoxetine	SERPINE1	0.12	18794724
40	Levothyroxine	SERPINE1	0.38	16075920
41	Vasopressin	SERPINE1	0.25	7482412
42	Cetrorelix	SERPINE1	1.05	16391860
43	Defibrotide	SERPINE1	0.84	12745658
44	Dexamethasone	SERPINE1	0.05	18285546
45	Hydrochlorothiazide	SERPINE1	0.11	11836266

## Discussion

In recent decades, hepatocellular carcinoma (HCC) incidence has increased in many countries. HCC is the main histological type of liver cancer and accounts for most liver cancer deaths [12]. Depression is a highly common mental disorder, and many patients do not respond adequately to existing treatments [13]. Chronic or early life stress is one of the key risk factors for depression. Epidemiological investigation found that the prevalence of depression in cancer patients is higher than that in the general population [14]. Cancer incidence is higher in patients with depression, and depression is linked to the increase in cancer-specific mortality, which is consistent with findings that depression and anxiety were significantly associated with increased risk of cancer (adjusted RR: 1.13, 95% CI: 1.06–1.19), cancer-specific mortality (1.21, 1.16–1.26), and all-cause mortality in cancer patients (1.24, 1.13–1.35) [15]. The estimated absolute risk increases (ARIs) associated with depression and anxiety were 34.3 events/100,000 person years (15.8–50.2) for cancer incidence and 28.2 events/100,000 person years (21.5–34.9) for cancer-specific mortality. However, so far, we have seen more clinical observational and epidemiological survey data. The genetic mechanism of the interaction between HCC and depression has not been determined. Here, we used bioinformatics to identify genes that are co-expressed in HCC and depression and to identify the biological process, cellular component, molecular function, and signaling pathways involved. Using DGIdb database, we identified drugs that may be used in both.

Our data show that HCC and depression share 199 genes. GO enrichment analysis shows that the top-6 biological processes are response to oxygen-containing compound, apoptotic process, programmed cell death, cell death, response to organic substance, and regulation of programmed cell death. Four of these are related to cell death/apoptosis. We hypothesize that before depression is apparent, the biological mechanism underlying the interaction between HCC and depression may be associated with cell death or apoptosis. Most anticancer therapies trigger apoptosis [16,17], and with molecular technology advances, anticancer drugs have targeted apoptosis with increasingly higher specificity. Exposition of pathophysiology of diseases from the gene level may better provide strategies for the development of new cancer treatments [18]. Li et al. proved experimentally that deletion or inactivation of genetic targeting of Survivin, an inhibitor of apoptosis, sensitized HCC cells to TNFα-induced cell death and significantly suppressed human and mouse HCC cells [19]. With regard to depression, we know that brain-derived neurotrophic factor (BDNF) is a growth factor, which influences neuronal survival, growth, and neuroplasticity. The tumor suppressor gene, Pdcd4 is an endogenous inhibitor of BDNF translation. BDNF expression changes are associated with severe depressive disorder. Animal studies show that blocking Pdcd4 in depression promotes BDNF expression [20], indicating that Pdcd4 may be a potential anti-depression target.

Four of the top-6 pathways are related to inflammation. At present, the mechanism of hepatocellular carcinogenesis due to viral hepatitis is clinically recognized [21]. Other studies show that T cells play a role in the immune escape mechanism of liver cancer [22]. For depression, mounting evidence shows a link between depression and inflammatory processes. Clinical trials have shown that the antidepressant effect of anti-inflammatory drugs can be used as an additional treatment or a single treatment, but important challenges remain [23–25].

Cluster-1 genes were inputted into GEPIA database to analyze their expression and 10-year survival rate in HCC. We concluded that IGF1, VEGFA, and SERPINE1 had statistical significance in the expression and 10-year survival rate between hepatocellular carcinoma group and normal group. Although the expression of VEGFA and SERPINE1 is low in HCC compared with normal group, survival rate in low expression group is higher than that in high expression group. This result is based on the statistics of the high and low expression groups of patients with liver cancer, which has nothing to do with normal people, so it is not contradictory. As for depression, histological analysis by Kondo et al. [26] showed that a serotonin type 3 receptor (5HT3R) and IGF1 are expressed in the same neurons in the subgranular region of the dentate gyrus of the hippocampus. 5HT3R regulates extracellular IGF1 levels in the hippocampus, a novel 5HT3R-IGF1 mechanism that differs from fluoxetine-induced responses. This provides a novel therapeutic target for depression, especially in depression patients with selective resistance serotonin reuptake inhibitors. However, genetic association analysis of polymorphisms in 10 genes belonging to the IGF-I system (IGF1, IGF1R, IGFBP1 to IGFBP7, and IGFBPL1) in the largest genetic databases for major depression (Psychiatric Genomics Consortium) revealed nominal associations with susceptibility to depression and treatment response, although results were not remain significant after multiple testing correction [27]. Cui [28] measured the VEGFA mRNA and protein in hippocampus tissues and sera from a rat model of depression and found that they were downregulated in hippocampi and blood of the depressed rats. Our results also showed low VEGFA expression in the tumor group, suggesting a new avenue for the pathology and treatment of HCC complicated by depression. Yi Y’s [29] study shows that SERPINE1 is upregulated in ovarian cancer patients with severe depression and worsens survival, but the mechanism is unknown. In patients with liver cancer complicated by depression, IGF1/VEGFA/SERPINE1 may play an important role in the pathogenesis and development of the disease, but the specific mechanism has not been clarified.

Finally, through the DGIdb database, 45 drugs were identified for VEGFA and SERPINE1. Because no drugs were identified for IGF1, its therapeutic value will be investigated in subsequent studies. The selected drugs can be validated via *in vitro* studies to provide reference values for clinical practice.

## Limitations

This study only explained the mechanism between hepatocellular carcinoma and depression using bioinformatics, and no experimental tests were done on the proposed hypothesis.

## Conclusion

In summary, we found the possible mechanism linking HCC and depression. By enriching the biological functions of related genes, we propose that the biological mechanism of the interaction between HCC and depression may be associated with cell death or apoptosis. These bioinformatics findings need experimental validation.

## Supplementary Material

Supplemental Material

## Data Availability

In this article, we used four databases and a web page mapping tool, as listed below: pubmed2ensembl (http://pubmed2ensembl.ls.manchester.ac.uk). VENNY (https://bioinfogp.cnb.csic.es/tools/venny/). DAVID (https://david.ncifcrf.gov/). STRING (http://string-db.orgis). GEPIA (http://gepia.cancer-pku.cn/). DGIdb (http://www.dgidb.org/search).

## Highlights

• Put forward the hypothesis that the mechanisms underlying the interaction between HCC and depression may be related to cell death or apoptosis.

• First explored relationship between HCC and depression was through data mining.

• Pointed pharmacological targets.
