# Supplementary figures and images for: Relationship between hepatocellular carcinoma and depression via online database analysis
Source: Bioengineered. 2021 May 7;12(1):1689–97. doi: 10.1080/21655979.2021.1921552 (PMC8806243; doi:10.1080/21655979.2021.1921552)

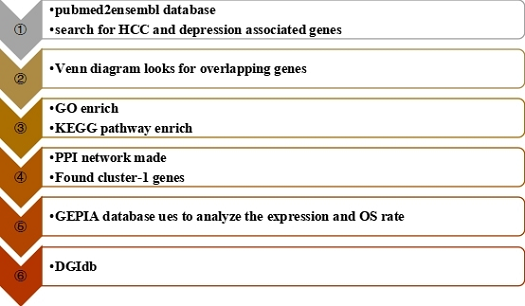

Supplement: Supplemental Material [file KBIE_A_1921552_SM9475.png]
